# 嵌合抗原受体T细胞与嵌合抗原受体调节性T细胞在自身免疫性疾病治疗中的研究进展

**DOI:** 10.3760/cma.j.cn121090-20250524-00242

**Published:** 2025-09

**Authors:** 信安 潘, 均 施

**Affiliations:** 1 中国医学科学院血液病医院（中国医学科学院血液学研究所），血液与健康全国重点实验室，国家血液系统疾病临床医学研究中心，细胞生态海河实验室，天津 300020 State Key Laboratory of Experimental Hematology, National Clinical Research Center for Blood Diseases, Haihe Laboratory of Cell Ecosystem, Institute of Hematology & Blood Diseases Hospital, Chinese Academy of Medical Sciences & Peking Union Medical College, Tianjin 300020, China; 2 天津医学健康研究院，天津 301600 Tianjin Institutes of Health Science, Tianjin 301600, China

## Abstract

自身免疫性疾病（AID）是一类由免疫系统错误地攻击自身组织而引起的疾病。然而现有免疫抑制治疗难以实现疾病的长期、无药物缓解，亟需更精准且长效的新型治疗策略。嵌合抗原受体T（CAR-T）细胞通过基因改造T细胞，能特异性识别并杀伤特定抗原表达的细胞，为AID的治疗提供了新的思路。本综述总结了CAR-T细胞及嵌合抗原受体调节性T细胞（CAR-Treg）治疗AID的免疫学机制，系统回顾了上述疗法治疗风湿性AID、免疫介导的神经系统AID及难治性自身免疫性溶血性贫血等多种AID的前沿进展。此外，我们还讨论了CAR-T / CAR-Treg细胞治疗AID的安全性，以及除CAR-T / CAR-Treg细胞之外其他可用于治疗AID的CAR细胞疗法。

## Abstract (en)

Autoimmune diseases（AID）are disorders in which the immune system mistakenly attacks the body's own tissues. However, current immunosuppressive therapies seldom achieve durable, drug-free remission, indicating the urgent need for therapeutic strategies that are both more precise and longer-lasting. Chimeric antigen receptor（CAR）-T cells are generated by genetically engineering T cells to specifically recognize and kill cells that express particular antigens, thereby providing a novel therapeutic approach for AID. This review summarizes the immunological mechanisms of CAR-T cells and -regulatory T cells（Treg）in treating AID, and systematically reviews the latest advances in applying these treatments to rheumatic AID, immune‑mediated neurological AID, and refractory autoimmune hemolytic anemia, among others. Further, we discuss the safety-related limitations of CAR-T /CAR-Treg treatment for AID, and outline other CAR-based cellular therapies that can be used to treat AID beyond CAR-T and CAR-Treg cells.

嵌合抗原受体T细胞（Chimeric antigen receptor-T cell, CAR-T细胞）治疗已经成为癌症免疫治疗的突破性方法，它可直接识别肿瘤相关抗原，而无需主要组织相容性复合物（Major histocompatibility complex, MHC）介导的抗原呈递[1]。目前该疗法在血液系统恶性肿瘤（如淋巴瘤和白血病）中展现出显著疗效[2]。美国食品药品监督管理局（FDA）已批准6种CAR-T细胞产品用于12种适应证，包括弥漫大B细胞淋巴瘤（Diffuse large B-cell lymphoma, DLBCL）、急性B淋巴细胞白血病（B-cell acute lymphoblastic leukemia, B-ALL）、套细胞淋巴瘤和滤泡性淋巴瘤等[3]。其中靶向CD19 CAR-T细胞疗法在B-ALL及滤泡性淋巴瘤中疗效显著[4]–[5]，而靶向CD30、CD20、CD7等新型靶点的疗法也在血液系统肿瘤的临床试验中应答率超过50％[3]。

自身免疫性疾病（Autoimmune diseases, AID）是一类由免疫耐受机制失衡所致的异质性疾病，按主要累及组织或器官可分为器官特异性AID（如重症肌无力、自身免疫性甲状腺疾病）和系统性AID［如系统性红斑狼疮（Systemic lupus erythematosus, SLE）、系统性硬化（Systemic scleredema, SSc）］[6]。目前，AID治疗主要依赖糖皮质激素、免疫抑制剂以及单克隆抗体（Monoclonal antibodies, mAb）。尽管糖皮质激素和免疫抑制剂能有效控制炎症反应，但长期使用易导致骨质疏松、感染风险增加及肾毒性等[7]–[8]。mAb类药物通过靶向清除T、B细胞和调节免疫耐受性发挥作用，包括抑制B细胞激活和自身抗体生成，清除异常B细胞。如阿仑单抗可耗竭异常T、B细胞并诱导调节性T细胞（Regulatory T cells, Treg）恢复免疫平衡[9]。然而，mAb难以清除组织驻留的自身反应性B细胞，而后者是驱动疾病活动的关键[10]。目前为止，AID尚未实现无药物缓解。与现有治疗方法相比，CAR细胞疗法凭借精准靶向、体内扩增和长效作用等优势，为突破AID治疗瓶颈提供了新方向。

本文回顾了CAR-T细胞以及嵌合抗原受体调节性T细胞（Chimeric antigen receptor regulatory T cells, CAR-Treg）治疗AID的机制，总结了在动物模型和临床试验中CAR-T/Treg细胞治疗AID的最新研究进展。此外，我们还讨论了CAR-T/Treg细胞治疗AID的安全性挑战及新型CAR细胞治疗策略。

一、CAR-T/Treg细胞治疗AID的机制

AID发生机制主要包括：①T细胞激活B细胞或直接杀伤靶细胞；②产自身抗体的B细胞增殖；③介导免疫耐受的Treg细胞数量减少或功能缺失；④补体系统过度激活或缺陷导致炎症和组织损伤[11]–[12]。这些机制为CAR-T/Treg治疗提供了干预靶点。

在AID中，自身反应性T细胞通过直接损伤组织或促进B细胞分泌自身抗体驱动疾病进展[13]。CAR-T细胞通过基因工程改造，呈递抗原识别结构域及激活信号，独立于抗原呈递细胞（Antigen-presenting cells, APC）发挥作用，与表达抗原的自身反应性T细胞结合后发挥细胞毒作用，有效抑制或消除这些病理细胞[14]。

逃避免疫耐受的B细胞通过分泌自身抗体、促炎因子或充当APC驱动自身免疫[15]。与mAb不同，CAR-T细胞不仅能靶向并清除此类异常B细胞，阻断自身抗体生成，抑制自身免疫反应和器官炎症，还可作为“活性药物”在体内长期存续[16]。

Treg细胞通过抑制CD4^+^和CD8^+^ T细胞、自然杀伤细胞及APC介导的促炎活性维持免疫平衡，其数量减少或功能缺陷时，会导致免疫失衡和AID发生[17]。Treg细胞作为一种独特的T细胞亚群，通过基因工程将CAR结构导入Treg细胞（CAR-Treg），改造后的CAR-Treg能通过选择性地靶向参与自身免疫病理的抗原，可增强其靶向自身免疫病理抗原的能力，抑制自身反应性T细胞的活化与增殖[18]–[19]。

二、CAR-T细胞治疗在AID小鼠模型中的临床前应用进展

目前，在多种AID小鼠模型中，CAR-T细胞治疗展现出显著的治疗效果，包括SLE、类风湿关节炎（Rheumatoid arthritis, RA）、格雷夫斯病（Graves' disease, GD）、原发性胆汁性胆管炎（Primary biliary cholangitis, PBC）、1型糖尿病（Type 1 diabetes mellitus, T1DM）。其作用机制主要依赖于靶向B细胞和T细胞，显著改善疾病症状并减少病理损伤。

（一）在风湿性AID小鼠模型中的CAR-T细胞治疗

1. 在SLE小鼠模型中的抗CD19 CAR-T细胞治疗：CD19作为B细胞激活、成熟和信号传导的关键分子，是SLE治疗的理想靶点[20]。研究表明，靶向CD19的第二代CAR-T细胞（含CD28共刺激域）在SLE小鼠模型中可持久清除CD19^+^ B细胞，抑制自身抗体产生，逆转靶器官病变，并延长小鼠寿命；此外，这些细胞在小鼠体内保持活性长达1年，表现出持久的免疫记忆功能[21]。Jin等[22]的研究进一步证实，含CD28或4-1BB共刺激域的抗CD19 CAR-T细胞在SLE小鼠中分别表现出有效的预防和治疗作用。

Doglio等[23]开发了一种FoxP3过表达的抗CD19 CAR-Treg细胞（Fox19CAR-Tregs）。在人源化小鼠模型中，Fox19CAR-Tregs显著减少了自体抗体产生，延缓了淋巴细胞减少，并恢复了淋巴器官的免疫系统组成。尽管小鼠的生存期较短，但该疗法通过打破导致自体免疫和组织损伤的恶性循环，有效保护了SLE相关靶器官。

2. 在RA小鼠模型中针对抗瓜氨酸化抗原抗体的CAR-T细胞治疗：RA的特征是在患者外周存在抗瓜氨酸化抗原的自身抗体，这些抗体通过识别自身蛋白中的瓜氨酸残基导致组织损伤[24]。Zhang等[25]开发了一种抗异硫氰酸荧光素（Fluorescein isothiocyanate, FITC）的CAR-T细胞，该细胞通过特异性识别FITC标记的瓜氨酸化抗原肽，清除RA中的自身反应性B细胞亚群。研究结果显示，该CAR-T细胞显著降低了小鼠关节炎的发病率，延缓了发病时间，减轻了疾病的严重程度，并减少了血清中的自身抗体和T细胞增殖反应。该临床前研究证实了CAR-T细胞治疗RA的有效性和安全性，为后续的临床转化研究提供了重要依据。

（二）在GD小鼠模型中的CAR-T细胞治疗

​GD是一种由促甲状腺激素受体抗体（Thyrotropin receptor antibodies, TRAb）介导的AID。TRAb通过激活促甲状腺激素受体，导致甲状腺激素过度分泌，从而引起甲状腺功能亢进[26]。目前，CAR-T细胞治疗在GD小鼠模型中显示出良好疗效。一项研究设计了一种靶向促甲状腺激素受体的CAR-T细胞，连续20 d静脉注射后，GD小鼠的甲状腺功能亢进显著逆转，表现为甲状腺体积显著减少，TRAb、游离甲状腺素以及游离三碘甲腺原氨酸水平恢复正常[27]。

（三）在PBC小鼠模型中针对高表达PD-1的CD8^+^组织驻留记忆T细胞的CAR-T细胞治疗

PBC是一种慢性自身免疫性肝病，其主要特征为肝内小胆管损伤和抗线粒体抗体升高。Zhu等[28]构建了一种PBC小鼠模型——DKO小鼠，发现DKO小鼠肝脏中存在高度活化并表达程序性细胞死亡受体-1（Programmed cell death protein 1, PD-1）的CD8^+^组织驻留记忆T细胞（CD8^+^ tissue-resident memory T cells, CD8^+^ Trm）；这些细胞具有强细胞毒性，可能在PBC的发生中起关键作用；研究人员开发一种靶向PD-1的CAR-T细胞，注射后DKO小鼠肝脏中PD-1阳性CD8^+^ Trm细胞数量显著减少，胆管周围炎症和纤维化明显缓解，肝脏炎症评分显著降低。

（四）在T1DM小鼠模型中的CAR-T/Treg细胞治疗

T1DM是一种自身免疫性糖尿病，其特征是自身抗体攻击胰腺β细胞，导致胰岛素绝对缺乏。一项研究构建了包含CD28和4-1BB共刺激域的mAb287 CAR-T细胞，能够特异性识别I-Ag7 MHC Ⅱ类分子与胰岛素B链9-23氨基酸片段[29]。在非肥胖型糖尿病（Non-obese diabetic, NOD）小鼠中，单次注射mAb287 CAR-T细胞后显著延缓了T1DM症状的出现，而未治疗的小鼠在18周龄前已发展为T1DM。CAR-T细胞的保护效应随时间减弱，提示单次输注无法完全预防T1DM的发展[29]。未来的研究还需提升CAR-T细胞的长期持久性以实现持续保护。

此外，Spanier等[30]开发了一种靶向胰岛素B链10-23肽段并由I-Ag7 MHC Ⅱ类等位基因呈递的CAR-Treg细胞（InsB-g7 CAR-Tregs细胞）。这种细胞通过减少BDC2.5 T细胞的增殖、IL-2产生以及降低树突状细胞上CD80和CD86的表达抑制T1DM的发生发展。在BDC2.5 T细胞诱导的NOD小鼠模型和野生型自发性NOD小鼠模型中，InsB-g7 CAR-Tregs细胞均显著降低了T1DM的发病率。

三、CAR-T细胞治疗在AID患者中的临床试验进展

迄今为止，已有临床试验报道了SLE及其他AID，如SSc、干燥综合征（​Sjögren syndrome, SS）、视神经脊髓炎谱系疾病（Neuromyelitis optica spectrum disorders, NMOSD）、重症肌无力（​Myasthenia gravis, MG）和多发性硬化（Multiple sclerosis, MS）的患者接受了靶向B细胞或浆细胞的CAR-T细胞治疗。

（一）风湿性AID

1. SLE：SLE的特征为多种自身抗体形成免疫复合物，沉积于组织并激活补体系统，引发炎症反应及多器官损伤[31]。Georg Schett团队报道了首例重度/难治性SLE患者接受自体抗CD19 CAR-T细胞治疗，患者的循环B细胞显著减少、抗体水平降低，蛋白尿改善[32]。Feng等[33]报告了12例难治性SLE患者接受CD19/ B细胞成熟抗原（B-cell maturation antigen, BCMA）CAR-T治疗的结果，患者在治疗后3个月B细胞水平恢复正常。Mei等[20]的一项临床试验结果显示，所有12例中重度活动性SLE患者接受自体CD19 CAR-T细胞输注后，疾病活动度显著降低，且安全性良好。

CAR-T细胞疗法在SLE及其伴发疾病治疗中也展现出巨大潜力。Wang等[34]研究显示，13例SLE合并狼疮性肾炎患者接受靶向CD19/BCMA CAR-T细胞治疗，9例患者在治疗后3个月内实现无药物缓解，11例肾功能改善。此外，该疗法在1例SLE合并DLBCL患者中展现出双重疗效[35]。CAR-T细胞治疗在儿童SLE患者中也取得进展。近期研究显示，2例12岁难治性SLE患儿接受抗CD19 CAR-T细胞治疗后症状显著改善，其中1例实现完全缓解[36]。

2. SSc：SSc是一种以自身免疫、血管病变、细胞外基质沉积和纤维化为特征的结缔组织病，可累及皮肤及内脏器官[37]。一例弥漫性SSc患者接受抗CD19 CAR-T细胞治疗后，肺部和心肌纤维化改善，疾病活动性降低[38]。另一例SSc合并非特异性间质性肺炎患者接受第三代CD19 CAR-T细胞治疗后，肺功能改善、皮肤纤维化减轻、自身抗体水平下降，CAR-T细胞在体内存活11个月[39]。Wang等[40]利用CRISPR-Cas9技术改造的抗CD19 CAR-T细胞治疗了2例难治性弥漫性皮肤SSc患者，在治疗后2周内实现B细胞完全清除，皮肤、肺部纤维化及心脏受累逆转，且未发生严重不良事件。

3. SS：SS是一种以免疫介导的唾液腺和泪腺损伤为特征的系统性AID[41]。Sheng等[42]报道了1例患者接受抗CD19 CAR-T细胞治疗后，抗核抗体和抗Ro-52抗体转阴，6个月内症状完全缓解，血清细胞因子水平恢复正常。

（二）免疫介导的神经系统疾病

1. NMOSD：NMOSD是一组以针对水通道蛋白4（Aquaporin 4, AQP4）抗体的产生为特征，激活补体系统，导致视神经和脊髓损伤的AID[43]。Qin等[44]报道12例复发难治性AQP4-IgG阳性NMOSD患者接受抗BCMA CAR-T细胞治疗，中位随访5.5个月，11例实现无药缓解且无复发，所有患者残疾状况和生活质量改善，血清AQP4-IgG水平下降。

2. MG：MG是一种T细胞依赖性、B细胞介导的AID，其发病机制为自身抗体攻击神经肌肉接头蛋白，损害神经肌肉传递，导致肌无力[45]。一项研究显示，14例Ⅱ~Ⅳ级MG患者接受靶向BCMA CAR-T细胞治疗后，MG严重程度评分显著降低，且未出现严重不良反应[46]。

CAR-T细胞疗法在MG伴发其他AID治疗中也具有巨大潜力。最近的一项临床试验中，1例重症MG伴抗环瓜氨酸蛋白抗体（Anti-Citrullinated Protein Antibodies, ACPA）阳性RA患者接受自体抗CD19 CAR-T细胞治疗后，ACPA水平下降，MG和RA症状缓解[47]。

3. 兰伯特-伊顿肌无力综合征（Lambert-Eaton myasthenic syndrome, LEMS）：LEMS为另一种由针对神经肌肉接头突触成分的自身抗体介导的AID。一项研究显示，2例重症MG合并LEMS患者接受自体抗CD19 CAR-T细胞治疗后，活动能力和生活质量显著改善，循环CD19^+^ B细胞水平恢复正常[48]。此外，Zhang等[49]首次使用靶向CD19/BCMA的双特异性CAR-T细胞治疗1例难治性MG患者，60 d达到完全缓解，MG严重程度评分下降，抗ACh抗体水平逐渐下降并转阴，且未出现症状复发或恶化。

4. MS：MS是一种以免疫细胞在中枢神经系统中的增殖和持续性神经炎症为特征的AID[50]。尽管免疫调节疗法在早期可抑制炎症，但通常无法阻止MS进展。Fischbach等[51]首次采用全人源CD19 CAR-T细胞治疗2例进展期MS患者，患者的扩展残疾状态量表评分保持稳定，未出现新的神经系统症状，提示该疗法对MS具有潜在疗效。

（三）难治性自身免疫性溶血性贫血（Refractory autoimmune hemolytic anemia, rAIHA）

AIHA的特征是B细胞产生自身抗体靶向红细胞，通过Fc受体介导的吞噬作用增加红细胞清除[52]。传统B细胞靶向治疗难以控制rAIHA。我们团队创新性采用抗CD19 CAR-T细胞治疗8例至少接受三线治疗失败的rAIHA患者，7例可评估患者均实现快速无治疗缓解，部分缓解和完全缓解的中位时间分别为15 d和57 d，1例患者在无治疗缓解6个月后复发。在安全性方面，患者仅表现为低级别的CRS，仅1例患者同时出现了1级CRS和免疫细胞相关的神经毒性综合征（Immune effector cell-associated neurotoxicity syndrome, ICANS），长期随访未见严重感染事件，安全性令人满意[53]。

四、CAR-T细胞治疗在AID的安全性挑战

CAR-T细胞治疗在AID中的安全性与在癌症治疗中存在显著差异。在B细胞恶性肿瘤治疗中，CAR-T细胞常引发急性毒性反应，包括CRS、ICANS以及免疫效应细胞相关的血液毒性[54]。然而，临床试验显示，AID患者接受CAR-T细胞治疗后的严重毒性发生率极低[32],[55]。但由于样本量有限，安全性数据需谨慎解读。

其次，淋巴清除预处理可能增加感染风险。在一项CAR-T细胞治疗复发/难治性ALL和非霍奇金淋巴瘤的临床试验中，患者从淋巴清除到CAR-T细胞输注这一过程中，约25％的患者发生感染[56]。目前，尚无CAR-T细胞治疗AID与预处理化疗导致严重感染的报道。

再者，CAR-T细胞治疗可能加重原有疾病或诱发新疾病。在一个SSc伴严重肺损伤的小鼠模型中，抗CD19 CAR-T细胞治疗在清除自身反应性B细胞的同时，加剧了肺纤维化并增加死亡率[57]。Chen等[58]报道了两例复发难治性DLBCL患者在CAR-T细胞治疗后3个月发生桥本甲状腺炎。此外，两例B细胞淋巴瘤患者接受CAR-T细胞治疗后，发生了CAR-T来源的T细胞淋巴瘤[59]。提示CAR-T细胞治疗可能通过未知机制诱发继发性AID或恶性肿瘤。

五、其他潜在基于CAR的细胞治疗AID

除了T细胞以及Treg细胞外，自然杀伤细胞（​Natural killer cells, NK cells）也可以作为CAR改造的候选细胞，并在AID治疗中展现出独特潜力。有研究人员设计了一种程序性死亡配体1（Programmed death-ligand 1, PD-L1）CAR NK-92细胞，可清除PD-1高表达的CD4^+^滤泡T细胞，并在狼疮模型小鼠中显著抑制记忆B细胞增殖、分化和免疫球蛋白分泌[60]。此外，CAR-NK细胞治疗在SS的临床试验中也显示出良好疗效。SS患者体内产生的抗LaA抗体能够识别并结合La/干燥综合征B抗原（La/SSB）蛋白的LaA表位[61]。为此，Meng等[62]设计了一种基于嵌合自身抗体受体的自然杀伤细胞CAAR-NK92MI，该细胞可特异性清除携带抗La/SSB自身抗体的B细胞。

六、展望

近年来，随着研究的深入和技术的提升，CAR细胞疗法在AID中显示出潜在的巨大治疗前景，但其研究仍处于早期阶段，其临床转化需要更深入的基础研究和大规模临床试验，以全面评估其安全性和疗效[63]。同时，CAR细胞疗法能否用于上述疾病以外的AID治疗也是未来重要的研究方向，能够为更多AID患者带来治愈的希望。
